# Finding the Missing Tuberculosis Patients

**DOI:** 10.1093/infdis/jix368

**Published:** 2017-11-06

**Authors:** Daniel P Chin, Christy L Hanson

**Affiliations:** 1 Bill & Melinda Gates Foundation, Seattle, Washington and; 2 Macalester College, St. Paul, Minnesota

**Keywords:** tuberculosis, patient pathway analysis, care seeking, patient centered care

Many of the key principles of tuberculosis (TB) control are credited to the work of Karel Styblo, the Dutch epidemiologist. Working in Tanzania, Styblo developed a set of registries to capture information on patients evaluated for TB and then used the registries to monitor and evaluate the performance of early TB control programs [1]. Given the success of his approach, Styblo popularized the use of the cohort analysis to evaluate treatment outcomes. In a cohort analysis, every patient started on treatment is accounted for and assigned a treatment outcome. This countered the common tendency to bias the reporting of treatment outcomes by excluding patients who failed to complete treatment.

Although the cohort analysis is widely used by national TB programs (NTPs) today, it does not capture information on TB treatment for every TB patient in a community. The traditional cohort analysis only contains treatment results for patients who are reported to the NTP; thus, the treatment outcomes of TB patients who are treated but not reported to the NTP are not captured. For example, the 2013 Indonesian national TB prevalence survey indicated that two-thirds of treated TB patients were not reported to the NTP [2]. Because those treated outside of the NTP generally have poorer treatment outcomes, a cohort analysis that excludes these cases overestimates the effectiveness of TB treatment in a community. In many countries, there is a high ratio of prevalent to notified TB cases, which suggests that there are, indeed, many unreported TB cases [3].

Beyond treatment, the principles of a cohort analysis can be applied more broadly to other aspects of TB care and prevention. It is equally important to use the rigor and thoroughness of a cohort analysis to evaluate the TB diagnostic process and the linkage to treatment. As the global community works to end the TB epidemic, the most pressing challenge is that >4 million TB patients each year—40% of all incident cases—are not reported to NTPs [3]. These “missing” patients are either undiagnosed or are diagnosed but not reported. We should prioritize finding these patients and linking them to effective treatment. Applying the principles of a cohort analysis to the entire process of care-seeking—and in doing so accounting for what happens to every patient—can help us better understand how to find these missing patients.

This supplement presents 2 approaches that allow us to more fully understand what happens to every TB patient from initial care-seeking to treatment completion. They help identify potential points of interventions to keep patients engaged in the diagnostic and treatment process. The first approach is the patient care cascade. This approach has been presented as an “onion” model (Figure 1), which considers drop-offs along several points in the patient care pathway. Naidoo and colleagues used data from laboratory services, electronic TB registries, published studies, and other sources to piece together different parts of the TB patient care cascade in South Africa: patients tested for TB, patients diagnosed with TB, patients started on TB treatment, and patients successfully treated [4].

**Figure 1.**
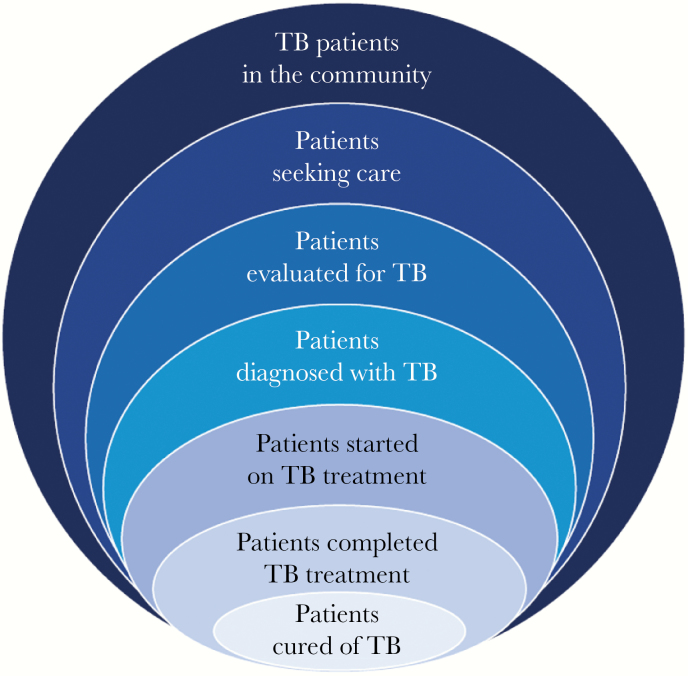
Onion model with progressively fewer patients at points along the patient care pathway. Abbreviation: TB, tuberculosis.

The results of the South African study demonstrate the importance of the patient care cascade. The traditional cohort analysis only tells us that 75% of TB patients started on treatment are successfully treated. Because the patient care cascade included all TB patients—not only those patients started on treatment—it appears that only 53% of TB patients in South Africa are successfully treated. As the South African NTP works to provide effective treatment for all its TB patients, these results suggest that the focus must go beyond more effective treatment. The NTPs also need to address the barriers to appropriate diagnosis and poor linkages to treatment after diagnosis, which are major contributors to the drop-offs in the patient care cascade. Based on these results, the South African NTP has focused its new National Strategic Plan on reducing drop-offs along the entire patient care cascade.

The second approach is the patient pathway analysis (PPA) [5]. The PPA begins where TB patients initiate care. Patient behavior and care are assessed at various points along the care-seeking pathway until the end of treatment. The analysis utilizes available population-based surveys to determine the degree of alignment between where patients initiate care, where TB services are available, and where patients are diagnosed or treated. The greater the misalignment between the location of care initiation and the availability of diagnostic and treatment services, the greater the likelihood that patients will drop out during the process of diagnosis and treatment. The PPA, therefore, provides valuable information on why there are “missing” cases.

Using the methods described in this supplement, we have carried out country-specific PPA to better understand the gaps contributing to the millions of missing cases globally. Figure 2 presents a combined 13-country PPA that includes the countries with the highest TB burden globally. It contains data from 5 country-specific PPA studies included in this supplement along with 8 other unpublished PPA studies. The methods for combining data from different PPA studies are described elsewhere [6]. In 2015, these countries contained 76% of incident TB cases, 64% of the multidrug-resistant TB cases, 65% of the human immunodeficiency virus–infected patients with TB, and—most importantly—92% of the missing TB cases in the world.

**Figure 2.**
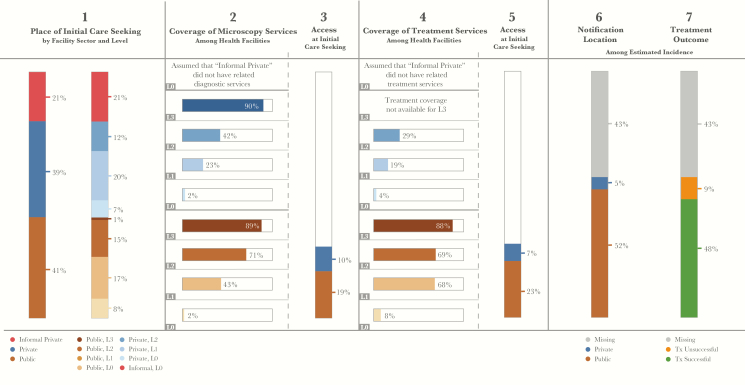
Combined 13-country patient pathway analysis. Countries include India, Indonesia, China, Nigeria, Pakistan, South Africa, Bangladesh, Philippines, Democratic Republic of the Congo, Ethiopia, Myanmar, Mozambique, and Kenya. In the formal public and private sectors, L0 refers to community level care and pharmacies; L1 refers to clinics and primary health care centers; L2 refers to lower-level hospitals; L3 refers to referral hospitals. In the informal sector, L0 refers to traditional healers and drug sellers.

This 13-country PPA provides important insights into what must be done to find the missing patients. First, it demonstrates that engaging the private sector is critical to find the missing patients. About 60% of all TB patients initiate care in the formal or informal private sector. However, <10% of all cases reported to the NTP are reported by the private sector. Thus, the vast majority of TB patients seeking care in the private sector are either not diagnosed or they are diagnosed but not reported to the NTP. Based on data from TB prevalence surveys and private sector drug sales, we can conclude that a significant number of TB patients are, indeed, treated in the private sector but just not reported [7, 8]. Because TB treatment in the private sector is highly variable and frequently of poor quality, it is critical to fully engage the private sector in the provision of quality TB care.

Second, the PPA showed that closing the current diagnostic gap is essential to find the missing patients. After >20 years of scaling up TB diagnostic services in high-burden countries, <30% of the facilities where TB patients initiate care can perform sputum smear microscopy. However, even fewer facilities have the capacity to conduct an Xpert test or refer a sample for Xpert testing [6]. This diagnostic gap is greatest in private facilities. But even in public facilities, smear microscopy is available in less than half of the primary healthcare clinics (L1) and less than three-quarters of district hospitals (L2). When patients reach a facility without TB diagnostics, they must travel to another facility at the same or a higher level of the health system to get a TB test. This delays the diagnostic process and increases the likelihood of drop-out along the care-seeking pathway.

Third, the PPA showed that improving the primary healthcare network and implementing the principles of universal healthcare are needed to find the missing cases. More than 70% of TB patients initiate care at the community (L0) or primary healthcare clinic (L1) level. A well-trained and properly incentivized cadre of community health workers is needed to recognize and evaluate patients with TB symptoms, and a well-functioning primary healthcare network is needed to provide proper TB testing, treatment, and referral services. The expansion of universal healthcare that extends essential services while mitigating financial shocks will allow all TB patients to access the services they need without incurring catastrophic health expenses [9].

The patient care cascade and the PPA are complementary approaches. Patient care cascades can quantify the magnitude of the gaps along the care pathway, while the PPA can determine the misalignment between the location of care initiation and the availability of diagnostic and treatment services. Both approaches are useful for developing a comprehensive understanding of why TB patients are missing and how to find them. The PPA is particularly useful to bring about patient-centered care and prevention—the first pillar of the World Health Organization END TB strategy [10]. As described in the country PPA studies, there are major subnational differences in care-seeking and availability of TB services [6]. Both approaches can be used at the subnational level to diagnose and treat the missing cases.

Although the patient cascade and the PPA approaches can be helpful, they also have limitations. Neither approach can determine the duration or factors contributing to delays in diagnosis or treatment [11]. They also do not capture information about the quality of available services [12, 13]. In addition, these approaches only provide us with information about specific steps along the care-seeking pathway, but not about what is happening between these steps. For example, the PPA indicates where patients initiate care but not how many additional facilities are visited before a diagnosis is made. Patients frequently follow a circuitous route to care, and understanding this route could help identify and implement effective interventions [14]. Thus, the patient cascade and PPA approaches should be used in conjunction with other data and information to obtain a more complete picture of the gaps contributing to the missing cases.

An important limitation of studies in this supplement is that none provided information about patients after their treatment ended. Our goal is to ensure that patients are permanently cured of their disease, free from subsequent disease relapse. In clinical trials using a standard 6-month regimen for drug-susceptible TB, 3%–5% of the patients have disease relapse after initially being cured [15, 16]. A higher percentage of patients treated under routine program conditions will relapse because treatment nonadherence is common. Unfortunately, NTPs do not collect reliable data on TB relapse. Even so, many countries report that 10% or more of their TB cases relapse after treatment success [3]. This highlights the importance of improving treatment adherence in high-burden countries. A more comprehensive view of the care cascade should include the continuum from disease onset to permanent cure without relapse, as was done in a recent care cascade study from India [17].

The global TB incidence is declining at only 1.5% per year [3]. At this rate, we will not come anywhere close to the target for TB in the Sustainable Development Goals (SDGs)—an 80% reduction in TB incidence by 2030. The 13-country PPA found that <50% of estimated TB patients have documented treatment success. Taking relapse into account, perhaps only 45% of patients are permanently cured. These sobering statistics help explain why the decline in TB incidence is so slow. The world will need better TB diagnostics, drugs, and vaccines to achieve the SDG targets. But while we await their development and availability, results from both the patient cascade and the PPA tell us there is a lot we can do today to accelerate the decline in TB incidence. We need to find the missing patients by understanding what happens to every TB patient, close the gaps along the patient care pathway, and provide effective, patient-centered care for all.

## Notes

***Supplement sponsorship.*** This work is part of a supplement sponsored by the Bill & Melinda Gates Foundation.

***Potential conflicts of interest.*** Both authors: No reported conflicts of interest. All authors have submitted the ICMJE Form for Disclosure of Potential Conflicts of Interest. Conflicts that the editors consider relevant to the content of the manuscript have been disclosed.
